# Adjustment method for microarray data generated using two-cycle RNA labeling protocol

**DOI:** 10.1186/1471-2164-14-31

**Published:** 2013-01-16

**Authors:** Fugui Wang, Rui Chen, Dong Ji, Shunong Bai, Minping Qian, Minghua Deng

**Affiliations:** 1 Center for Quantitative Biology, Peking University, Beijing, 100871, China; 2 LMAM,School of Mathematical Sciences, Peking University, Beijing, 100871, China; 3 School of Life Science, Peking University, Beijing, 100871, China; 4 Center for Statistical Sciences, Peking University, Beijing, 100871, China

**Keywords:** Microarray, Gene Expression, IVT, Two cycle amplification, RNA Degradation, Bias correction, Clustering

## Abstract

**Background:**

Microarray technology is widely utilized for monitoring the expression changes of thousands of genes simultaneously. However, the requirement of relatively large amount of RNA for labeling and hybridization makes it difficult to perform microarray experiments with limited biological materials, thus leads to the development of many methods for preparing and amplifying mRNA. It is addressed that amplification methods usually bring bias, which may strongly hamper the following interpretation of the results. A big challenge is how to correct for the bias before further analysis.

**Results:**

In this article, we observed the bias in rice gene expression microarray data generated with the Affymetrix one-cycle, two-cycle RNA labeling protocols, followed by validation with Real Time PCR. Based on these data, we proposed a statistical framework to model the processes of mRNA two-cycle linear amplification, and established a linear model for probe level correction. Maximum Likelihood Estimation (MLE) was applied to perform robust estimation of the Retaining Rate for each probe. After bias correction, some known pre-processing methods, such as PDNN, could be combined to finish preprocessing. Then, we evaluated our model and the results suggest that our model can effectively increase the quality of the microarray raw data: (i) Decrease the Coefficient of Variation for PM intensities of probe sets; (ii) Distinguish the microarray samples of five stages for rice stamen development more clearly; (iii) Improve the correlation coefficients among stamen microarray samples. We also discussed the necessity of model adjustment by comparing with another simple adjustment method.

**Conclusion:**

We conclude that the adjustment model is necessary and could effectively increase the quality of estimation for gene expression from the microarray raw data.

## Background

Gene expression microarrays are widely utilized for transcriptome analysis of biological samples from different treatments or different phenotypic groups. However, due to the difficulty of extracting sufficient amount of starting mRNA or total RNA from biological materials, such as rice stamen, it is usually impossible to detect the amounts and sequences of mRNA directly. Therefore, amplification of mRNA sample is necessary before performing microarray experiment to detect the fluorescence signals of mRNA [1-4]. RNA linear amplification technology, based on T7 RNA polymerase and in vitro transcription (IVT) (Affymetrix, Santa Clara, CA, USA), gradually becomes a mostly used protocol for target preparation in microarray experiments [5], mainly for three reasons. First, it reduces the required amount of starting materials to 1 ∼100 ng of total RNA. Second, the bias from the amplification is smaller than typical PCR for DNA. Third, it only requires mRNA or total RNA, rather than DNA, hence leads to a wider application [6].

**Figure 1:**
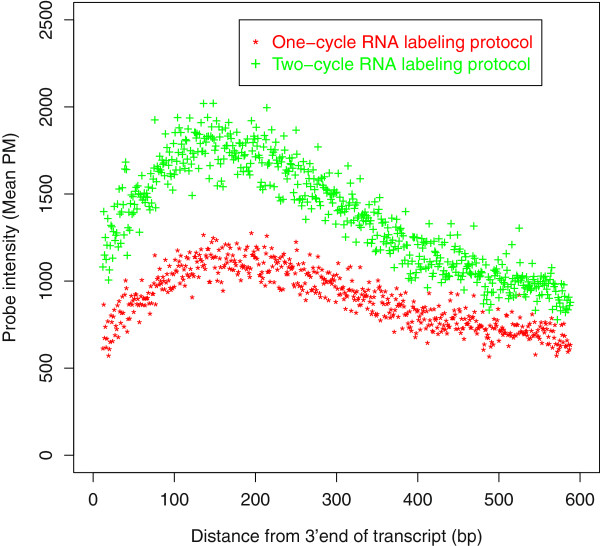
**Correlation between position and intensity of probes.** Correlation between position and intensity of probes for present probe sets in one-cycle and two-cycle amplification microarray data. X-axis is the distance of probe (the *13th* nucleotide) from 3’end of its corresponding transcript, while Y-axis is the mean PM intensity of probes at each position. Microarray data generating using one-cycle (red), two-cycle (green) RNA labeling protocols are plotted respectively. We only plot the probes within 600bp of 3’ end on transcripts (account for 98.68*%* of the present probesets). It’s obvious that variation in two cycle is larger and the bias is more serious.

However, two-cycle amplification produces larger bias associated with higher amplification efficiency [7,8]. In order to monitor the bias associated with RNA amplification, we generated two types of rice microarray data using both One-Cycle and Two-Cycle Eukaryotic Target Labeling Assay from Affymetrix (Santa Clara, CA, USA). Both showed the same decreasing trend of probe intensity near 3’ end and 5’ end of transcripts (See Figure 1 for more details).

It is also notable that, for mRNA transcripts, the decreasing trend of the two-cycle labeling is heavier than that of the one-cycle labeling, which implies that we are probably misled. Three possible reasons may account for this phenomenon. The first one is the degradation of transcript due to RNA’s instability, especially in 16 hour’s *in vitro* transcription (IVT) stage during the amplification process. Another reason might be the usage of random primers in the start of the second round amplification, which concludes uncompleted reverse transcription from aRNA to cDNA. The third reason may be the saturation of probe spot on microarray chip. Therefore, it is necessary to revise the microarray data generated with two-cycle RNA amplification before using it to perform further analysis, such as detecting differentially expressed genes, constructing co-expression gene network and so forth.

A few works before have also mentioned the problem of RNA degradation during two-cycle labeling [7-12]. However, there is almost no universally applicable solutions reported to deal with this problem. In this study, we proposed a statistical framework to model the process of mRNA two-cycle linear amplification, and established a linear model to revise the expression intensity at probe level. Probe level correction in this study means bias correction for intensity of Perfect Match probe (PM) if there is no special instruction.

This paper proceeds as follows. In the second section, we described three types of microarray experiments and a Real Time PCR experiment to validate the degradation effect. After that, we proposed a probabilistic model for probe level adjustment, and the parameters are estimated by MLE. In the third section, we displayed the bias existed in microarray experiment and validated the bias by Real Time PCR. Then we discussed the effects that might bring bias, especially the degradation effect in the RNA two-cycle labeling protocol. By applying our model to two stamen microarray data sets, we showed that our model adjustment obtained a significant improvement for the quality of microarray raw data. Finally, we discussed the necessity of our model adjustment and a possible application to RNA-seq data in the last section.

## Methods

### Materials and Methods

In this chapter, we first briefly introduced three kinds of microarray data of rice, and then described the designed Real Time PCR experiment to revalidate transcriptional degradation. For brevity, we refer to these three data sets as Data Set 1, Data Set 2 and Data Set 3, respectively. Plant materials as well as sample collection and total RNA isolation method are the same in three data sets. Data Set 1 was designed to show the phenomena of RNA degradation and to estimate parameters in the model. Data Set 2 was designed to validate the reproducibility of the bias from RNA degradation and to determine whether the bias is sample specific. Data Set 3 was applied to validate the efficiency of our adjusting model. After that we established a probabilistic model to estimate the extent of bias caused by RNA degradation during two-cycle linear amplification and attempted to correct for this bias. As mentioned above, the reason why bias is introduced is mainly due to the using of random primers and RNA degradation, on which our model is based. The saturation effect of micoarray chip is beyond our consideration.

#### Plant materials

Rice (O. sativa L. ssp. japonica cv. Zhonghua 15) seeds were soaked in water at 30°*C* for about one week. And then seedlings were transplanted in plastic pots and cultured at 30 ±2°*C* under 11 hours day/13 hours night cycle.

#### Microarray Data Set 1

The rice embryos were obtained from the seeds soaked in water for 2-3 days. The third mature expanded leaves were collected when they had just fully expended. Rice seedlings were harvested when they were 2-3 cm in height. The panicles were collected when they were 0.6-4 cm in length. Total RNA was isolated separately from rice embryos, the third mature leaves, the fifth mature leaves, seedlings and panicles with TRIzol Reagent from Invitrogen Life Technologies.

To obtain microarray data covering almost all genes in rice, total RNA from different samples described above was mixed into a big pool called Pre-amplified mRNA sample (PAM) by almost equal magnitude. Eight subgroups were separated from the purified PAM. The half of them were amplified and labeled by the One-Cycle Eukaryotic Target Labeling Assay from Affymetrix (Santa Clara, CA, USA). The resulting samples were called One-Cycle cRNA samples (OCS). The other half were amplified and labeled by the Two-Cycle Eukaryotic Target Labeling Assay from Affymetrix (Santa Clara, CA, USA). The resulting samples were called Two-Cycle cRNA samples (TCS). All experimental procedures strictly followed instructions specified in the Affymetrix GeneChip Expression Analysis Technical Manual.

#### Microarray Data Set 2

This data set contained microarray data from two different samples, including the third leaf primordium and the third mature leaves of rice. Total RNA was extracted from the two samples. And then each of them was separately amplified by two methods, the One-Cycle Labeling Assay and the Two-Cycle Labeling Assay (Affymetrix, Santa Clara, CA, USA). Two replicates were applied for each Assay. Totally there are 8 hybridization samples for the following microarray experiments.

#### Microarray Data Set 3

This data set included 21 slides of Affymetrix microarray. There are 7 different stages from rice, including the third leaf primordium, the third mature leaves, and stamen samples at stage 2, 3, 4, 5 and 6 (Stages are partitioned by the development of Stamen) [13,14]. Biological replicates are applied 3 times for each of them. All the 21 mRNA samples are amplified with Two-Cycle Labeling Assay (Affymetrix, Santa Clara, CA, USA).

All amplified RNA samples were hybridized with the Affymetrix GeneChip Rice Genome oligonucleotide arrays, which is widely used for rice gene expression analysis. The.CEL files are available at http://www.math.pku.edu.cn/teachers/dengmh/RiceTCAC/index.html.

### Validation of RNA degradation by Real Time PCR

To validate the degradation trend mentioned above, we developed a strategy by applying Real Time PCR to several transcripts. Real Time PCR were carried out using the same RNA samples as that of Data Set 1, including Pre-amplified mRNA samples (PAM), One-Cycle cRNA samples (OCS), and Two-Cycle cRNA samples (TCS).

In brief, primers were designed for transcripts using Real Time PCR Primer Design tool (http://www.genscript.com/cgi-bin/tools/primerâˆ–_genscript.cgi?op=standard). Each primer was further confirmed by dissociation curve analysis after the PCR reactions. First strand cDNA was synthesized by reverse transcription using 1 *μ*g of total RNA in 20 *μ*l of reaction volume using SuperScriptTM III Reverse Transcriptase from Invitrogen Corporation. Diluted cDNA samples were used for Real Time PCR analysis with 100 nM of each primer mixed with SYBR Green PCR master following manufacturer’s instructions. The reactions were carried out in Optical 96-Well Fast Plate on the 7500 Fast System (Applied Biosystems, USA).

We designed several pairs of primers locating from 5’end to 3’end of a transcript. The target of each pair of primers was called amplicon. Then we used the pairs of primers to carry out Real-time PCR with PAM, OCS and TCS separately. The Schematic diagram of Real Time PCR experiments is shown in Additional file 1: Figure S3. In order to remove the amplification efficiency of different amplicons, expression value of amplicon in PAM was served as reference. We calculated the relative expression values of the ith amplicon in jth sample *R*_*ij*_ (see Equation 1).

(1) $$R_{\text{ij}}=2^{-(CT_{\left(i,j\right)}-CT_{\left(i,\text{PAM}\right)})}\;j=\{\text{OCS},\text{TCS}\}$$

Where CT (cycle threshold) is the number of cycles required for fluorescent signal to reach the threshold. The larger CT is, the less the amount of RNA is. For a fixed j, among all the amplicons of a transcript, amplicon with the maximum relative expression value (*MAX* (*R*_*ij*_)) located in the region with minimal impact by degradation and amplification. And *MAX*(*R*_*ij*_) was closest to the actual expression level of the transcript. So we calculated the *DP*_*ij*_ of each amplicon relative to the *MAX* (*R*_*ij*_) to make clear the impact of degradation and amplification on different regions of transcript:

(2) $$DP_{\text{ij}}=1-\frac{R_{\text{ij}}}{\text{MAX}(R_{\text{ij}})}\;j=\{\text{OCS},\text{TCS}\}$$

*DP*_*ij*_ is a number between 0 and 1, where 0 means no degradation effect and 1 indicates the amplicon is completely degraded. The closer *DP*_*ij*_ is to 0, the less the region of ith amplicon is affected by amplification in jth sample, and vice versa.

### Model Adjustment for RNA amplification

**Figure 2:**
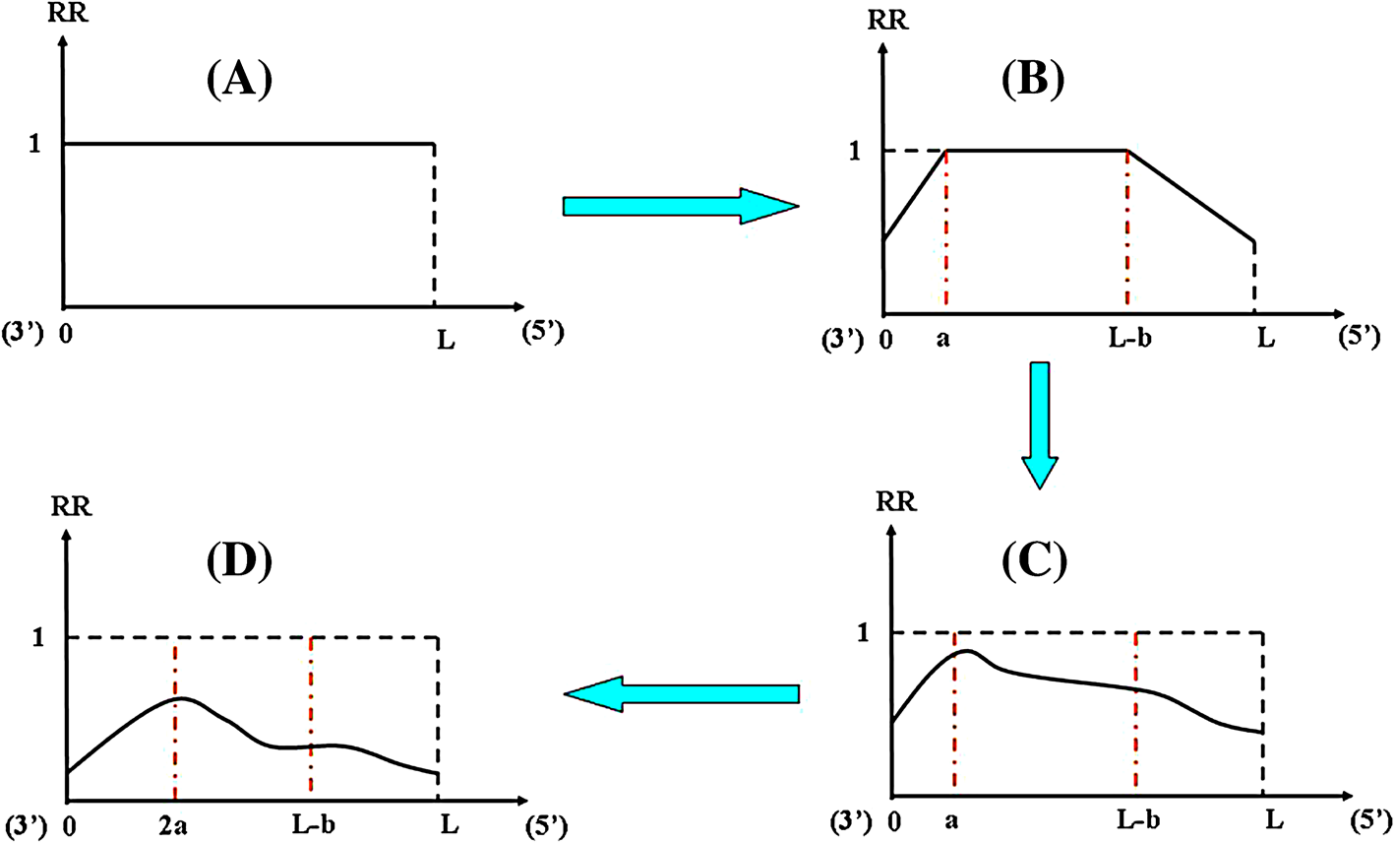
**Illustration for the process of mRNA transcript degradation during RNA two-cycle amplification.** The transcript of a probe set becomes shorter 3 times from the beginning to the end of two-cycle amplification. x-axis is the distance of probe from the 3’end of transcript (bp). y-axis represents the retaining probability of each position after the *i*th-cycle. *L* represents length of a transcript. *a* and *b* are degrading limits from 3’end and 5’end.

According to the two-cycle linear amplification protocol, for each RNA transcript, it might have been shortened three times from the beginning to the end of two-cycle amplification (See Figure 2, details see manual in [7]), which are: (i) RNA degradation during the IVT of first cycle amplification; (ii) Introducing of random primer in the second cycle; and (iii) RNA degradation during the IVT of second cycle amplification.

To better understand the model, we introduce the notation. To simplify calculation, we first assume that RNA is degrading at an even speed, regardless the sequence effect, e.g. GC content. Let 0 and *L* (base pair, bp) be the positions of 3’ end and 5’ end of a transcript before amplification, where *L* is the length of the transcript. Let *A*_*i*_ and *B*_*i*_ be the position of the new 3’ and 5’ end after the *ith* shorten (*i* = 1,2,3). It’s obvious that *A*_0_ = 0, *B*_0_ = *L*. (i) After the first cycle amplification, based on the uniform assumption of RNA degradation, we further assume *A*_1_ ~ *U*[*A*_0_,*A*_0_ + *a*] and *B*_1_ ~ *U*[*B*_0_ − *b*,*B*_0_], where *a*,*b* are the degradation limits of 3’ end and 5’ end separately. (ii) In the second cycle of amplification, 3’ and 5’ ends varies more complicated. As random primer is used in the step of the first strand cDNA synthesis from antisense RNA produced in the first cycle of amplification, which may result in incomplete synthesis. 5’ end is decreased while 3’ end remains the same. Therefore, the position of new 3’ end *A*_2_ is the same as *A*_1_, while the position of new 5’ end *B*_2_ ~ *U*[*A*_1_,*B*_1_], as random primer can bind to any position of the cRNA. (iii) As the same as in the first cycle, degradation takes place at both 3’ end and 5’ end in the IVT steps of the second cycle amplification. So it’s similarly concluded that *A*_3_ ~ *U*[*A*_2_,*A*_2_ + *a*] and *B*_3_ ~ *U*[*B*_2_ − *b*,*B*_2_], where *a*,*b* are the same as in the first cycle.

As most RNA transcripts are more than 200 bp, we utilized integral to substitute sum for calculation. Then we could obtain the joint distribution of (*A*_*i*_,*B*_*i*_),*i* = 1,2,3.

 i) The joint distribution of *A*_1_ and *B*_1_ is:

(3) $$\begin{array}{ll} F_{1}(x,y) & =P(A_{1}\leq x,B_{1}\leq y)\\ & =\frac{x(y+b-L)}{\text{ab}},(x\in [0,a],y\in [L-b,L]) \end{array}$$

ii) The joint distribution of *A*_2_ and *B*_2_ is:

(4) $$\begin{array}{lll} F_{2}(x,y) & =P(A_{2}\leq x,B_{2}\leq y)\; & \;\\ & =\overset{a}{\underset{0}{\int }}dx^{{}^{\prime }}\overset{y}{\underset{L-b}{\int }}P(A_{2}\leq x,B_{2}\leq y|A_{1}\; & \;\\ & =x^{{}^{\prime }},B_{1}=y^{{}^{\prime }})p_{1}(x^{{}^{\prime }},y^{{}^{\prime }})dy^{{}^{\prime }}\; \end{array}$$

where

(5) $$p_{1}(x^{{}^{\prime }},y^{{}^{\prime }})=\frac{\partial^{2}F_{1}(x^{{}^{\prime }},y^{{}^{\prime }})}{\partial x^{{}^{\prime }}\partial y^{{}^{\prime }}},$$

$$\begin{array}{ll} P & \left(A_{2}\leq x,B_{2}\leq y|A_{1}=x^{{}^{\prime }},B_{1}=y^{{}^{\prime }}\right)\\ & \;=\left\{\begin{array}{l} \frac{y-x}{y^{{}^{\prime }}-x},\;\text{for}\;\;x=x^{{}^{\prime }}\\ 0,\;\;\;\text{for}\;\;x\neq x^{{}^{\prime }} \end{array}\right) \end{array}$$

iii).The joint distribution of *A*_3_ and *B*_3_ is:

(6) $$\begin{array}{lll} F_{3}(x,y) & =P(A_{3}\leq x,B_{3}\leq y)\; & \;\\ & =\overset{x+a}{\underset{x}{\int }}dx^{{}^{\prime }}\overset{y}{\underset{y-b}{\int }}P(A_{3}\leq x,B_{3}\leq y|A_{2}\; & \;\\ & =x^{{}^{\prime }},B_{2}=y^{{}^{\prime }})p_{2}(x^{{}^{\prime }},y^{{}^{\prime }})dy^{{}^{\prime }}\; \end{array}$$

where

$$\begin{array}{ll} p_{2}(x^{{}^{\prime }},y^{{}^{\prime }})=\frac{\partial^{2}F_{2}(x^{{}^{\prime }},y^{{}^{\prime }})}{\partial x^{{}^{\prime }}\partial y^{{}^{\prime }}}, & \; \end{array}$$

(7) $$\begin{array}{ll} P(A_{3}\leq x,B_{3}\leq y|A_{2}=x^{{}^{\prime }},B_{2}=y^{{}^{\prime }})\; & \;\\ \;=\frac{\left(x-x^{{}^{\prime }})(y+b-y^{{}^{\prime }}\right)}{\text{ab}}\; \end{array}$$

Thus *F*_3_(*x*,*y*) can be rewritten out piecewise (shown in Additional file 1: Formula F1). Then, for each transcript’s products, we could estimate a Retaining Rate function *p*_*i*_(*z*), *i* = 1,2, which states a probability indicates how probable the the nucleotide on the position of *z* (bp) retains after the incomplete synthesis in the *ith* cycle amplification. Here, *z*, in [0,*L*], represents the distance of a nucleotide on the transcript away from its 3’ end. After the first cycle amplification, the Retaining Rate function *p*_1_(*z*), can be easily calculated from the distribution of 3’ and 5’ end:

(8) $$p_{1}(z)=\left\{\begin{array}{ll} \frac{z}{a}, & \;\text{for}\;\;0\leq z\leq a\\ 1, & \;\text{for}\;\;a<z<L-b\\ \frac{L-z}{b}, & \;\text{for}\;\;L-b\leq z\leq L \end{array}\right)$$

But for the second cycle amplification, the Retaining Rate function *p*_2_(*z*) is complicated piecewise, associated with *F*_3_(*x*,*y*). We calculated it by integrating with R software.

### Adjustment of biased signal

For each transcript, there is a probe set containing proximately 11 probes (99.58% of 57381 probe sets for rice microarray) on microarray to detect its the expression signal. We first define a Retaining Rate for a position on transcript, which is a probability indicates how probable the position of the probe on transcript remains after two-cycle amplification. Before the first step of two-cycle amplification, the Retaining Rate for each probe is 1, when amplification is over, the retaining rate for probe at *z* is *p*_2_(*z*) (Note that *z* refers to distance of the middle nucleotide of probe from 3’ end of the transcript). For the *kth* poly(A) RNA, the ideal intensity of *ith* probe after *jth* cycle, j = 1,2, is *IPS*_*jk*_; and the observed intensity (PM intensity) for *ith* probe at position *z*_*ik*_ is *IP*_*ijk*_. Then we can get *IP*_*ijk*_ as a function of *IPS*_*jk*_:

(9) $$IP_{\text{ijk}}=\text{IP}S_{\text{jk}}\times P_{j}(z_{\text{ik}})+\varepsilon_{\text{ijk}}$$

For determined *j*, *k* and all *i*, $\varepsilon_{\text{ijk}}∼N(0,\sigma_{\text{jk}}^{2})$, where $\sigma_{\text{jk}}^{2}$ is unknown. Then least square estimation becomes maximum likelihood estimation.

Thus, the maximum likelihood estimation for *IPS*_*jk*_ is:

(10) $$\text{IP}S_{\text{jk}}=\frac{\underset{i}{\sum }(IP_{\text{ijk}}\times p_{j}(z_{\text{ik}}))}{\underset{i}{\sum }p_{j}^{2}(z_{\text{ik}})}$$

where *i* = 1,2,…*n*_*j*_, *n*_*j*_ is the number of probes in the *jth* probe set.

Although the above model only uses PM of probe for training the parameters, it can also be used to adjust MM intensity, as Retaining Rate represents the probability of the nucleotide of each position on transcript to be remained after two-cycle amplification. As our model only correct for the bias caused by degradation during amplification at probe level, the corrected intensity didn’t perform the background correction or normalization at probe set level. We think it necessary to perform a further pre-processing to adjust for bias introduced by cross-hybridization or other factors. After adjusting for PM and MM, we could apply many existing popular normalization methods, such as PDNN [15], dchip [16] and RMA [17] to preprocess the modified microarray data before further investigation. In this study, we choose PDNN as it is more powerful by considering the sequence binding information [18,19].

## Results

### Degradation trends of transcripts

In the beginning of the first cycle amplification, a transcript is complete poly(A) RNA, whose cap in 5’ ends and tail in 3’ ends to some extent protect the RNA sequence. Thus, degradation of the transcript is relatively slow in this process. However, when the second cycle begins, as the RNA cap in 5’ end and tail in 3’ end have degraded or almost degraded, the degrading effect becomes heavier and thus leads the RNA sequence to be shorter [7]. Besides, using random primer also leads the RNA sequence to become shortened directly. As a result, it implies that the key effect of degradation may hide in the second cycle. Combining with the protocol of the Two-Cycle Labeling Assay (Affymetrix, Santa Clara, CA, USA), we postulated that there may be three possible reasons for the bias listed as follows. The first effect is RNA degradation in amplification process. With regard to RNA’s instability comparing with DNA, degradation would probably take place in the tube of RNA amplification, even in the initial RNA period which the degraded loss would amplify in the end. Second, random primers used in the second cycle have dubitable uncertainties that, unlike the oligo (dT) primer, can bind not only to 3’ poly(A) end of RNA, but also to the middle or the other end of transcript, concluding cDNA’s uncompleted reverse transcription from amplified RNA, which leads greater bias after the second cycle amplification. Third, in some cases, real expression intensity on some probe spots are underestimated, because of the saturation of the microarray chip.

Regardless the decaying speed of a mRNA varying by different ribonucleic acids and its secondary structures, we assume that a ribonucleic acid on mRNA degrades linearly relied on the distance to an end, either 3’ end or 5’end. Also, we assume that there exists a degrading limit, behind which degradation seldom takes place. We are clear that mRNA degrades from both 5’ and 3’ ends. In one pathway, mRNA shortening is followed by removal of the 5’ cap structure. Decapping gives a 5’-to-3’ exonuclease access to degrade the remainder of the mRNA [9]. In the other pathway, poly (A) shortening is followed by 3’-to-5’ digestion by a complex of exonucleases named the exosome. The exosome is distinct from the exonuclease that removes the poly (A) tail [9]. But poly (A) shortening is absent in microarray data, for hybridization losing the poly (A) fragments. Thus, degradation of 3’ end in microarray data is slighter and more easily treated. Another reason why we first consider degradation of 3’ end is that 5’ end’s decline attributes not only to decaying, but also to incomplete reverse transcription by random primers. For all 631066 probes of 57381 probe sets of rice microarray, we collected their distance away from 3’ end of transcript (Each probe represented by its middle nucleotide), and plotted the number of probes at the same position to show its distribution (Additional file 1: Figure S1). It’s obvious that most probes are designed to have distances less than 577 bp from 3’ end, and the number decreases between 30 bp and 577 bp. A similar gradient for 235700 probes of 21407 present probe sets (selected by MAS5.0) is shown in Additional file 1: Figure S1. For present probes, we calculated the mean of PM intensities of probes at the same distance (Figure 1). Two lines, respectively, are plotted from the same biological sample, microarray data using one-cycle (Green) and two-cycle (Red) amplification. It is obvious that, with the same trend, the two lines increase before the left of 133 bp from 3’end, and then decrease until 577 bp. No apparent pattern appears after that, because low number of probes at the same position breaks stability, i.e, there are a small number of probes on transcript further than 600 bp from 3’end, which make it to be of no statistical significance. From Figure 1, it’s obvious that variation of mean intensity in the two cycle labeling is much larger and the bias trend is much more serious than the one cycle labeling. Besides, leaf samples using the Affymetrix one-cycle and two-cycle RNA labelling protocols with the Rice microarray in Data Set 2 shows similar degradation trend (See Additional file 1: Figure S2), which demonstrates that this situation is technique specific rather than sample specific.

### Result of Real Time PCR

**Figure 3:**
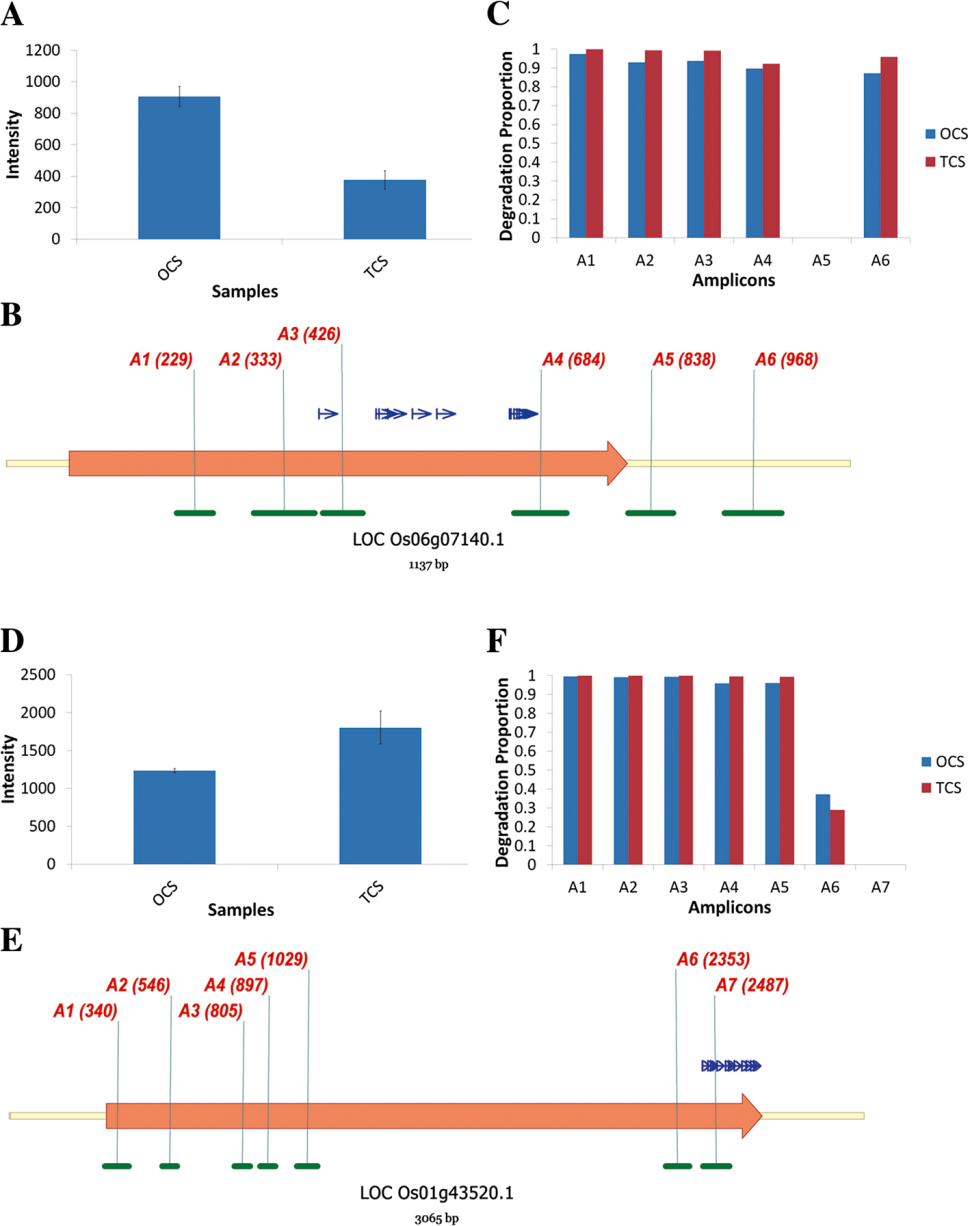
**Real time PCR results for two genes.** Real time PCR results confirmed correlation between position and intensity of probes afteramplification. **(A-C)** Data for LOC Os06g07140.1. **(A)** The intensity of LOC Os06g07140.1 decreased dramatically in Two-Cycle cRNA sample (TCS) comparing with One-Cycle cRNA sample (ONS) in our microarray experiment. **(B)** Schematic diagram of LOC Os06g07140.1. Yellow line, cDNA of the gene; Big orange arrow, coding sequence (CDS) of the gene; Green line section **(A1-A6)**, designed amplicons in Real Time PCR experiments; Numbers in the brackets were the starting point of amplicons from 5’ end; Blue narrow arrow, designed probes on the microarray. Their starting points (unlabeled in the figure) were 421, 498, 502, 514, 547, 580, 678, 680, 684, 688, and 691. **(C)** Degradation Proportion (DP, see details in Methods section) of amplicons **(A1-A6)** showed in **(B)**. DP decreased along with distance from the 5’ end and increased when it was too close to 3’ end because of degradation and random effect. **(D-F)** Data for LOC Os01g43520.1. **(D)** The intensity of LOCOs01g43520.1. **(E)** Schematic diagram of LOCOs01g43520.1. **(A1-A7)** were designed amplicons. The probes’ starting points on microarray were 2485, 2505, 2515, 2537, 2568, 2579, 2599, 2627, 2642, 2658, and 2669. **(F)** DP of amplicons **(A1-A7)** showed in **(E)**. Because there was no amplicon too close to 3’ end, DP didnt increase again.

After carrying out Real Rime PCR with primers mentioned above, we calculated the Degradation Proportion (DP) of each amplicon. The details about DP were described in method section. Briefly, the closer *DP*_*ij*_ was to 0, the less the region of ith amplicon was affected by degradation and amplification in the jth sample, and vice versa. Because of degradation and amplification, we could expect that the DP of amplicons near the 5’ end to be higher. And the further away amplicon was from 5’ end, the lower the DP was. But when amplicons were very close to 3’ end of transcript, the DP would increase again because of 3’ degradation. According to the annotation from Affymetrix company, LOC_Os06g07140 (Gene Nomenclature from MSU Rice Genome Annotation Project) was represented by probe set Os.4216.1.S1_a_at on the GeneChip Rice Genome Array. And its intensity decreased dramatically in TCS comparing with OCS (Figure 3, A). We designed 6 amplicons (A1-A6) to its transcripts(Figure 3, B, green line). As we expected, DP of A1 to A4 was approximately to 1, decreased along with the position from 5’ to 3’ end. A5 was close to 3’ end and had the minimal DP. It meant that if the probes were designed at this position, the intensity would be the nearest to the actual expression level. Interestingly, the DP of A6 had increased despite its position was very close to the 3’ end. It demonstrated that degradation from 3’ end also took effect (Figure 3, C). Probe set Os.4216.1.S1_a_at comprised 11 probes, and all of them located at the region of 421-691bp from 5’ end. It could explain why the intensity of Os.4216.1.S1_a_at in TCS was underestimated. We also selected another gene, LOC_Os01g43520.1. It was represented by probe set Os.652.1.S2_a_at. Its intensity exhibited opposite profile comparing to Os.4216.1.S1_a_at in OCS and TCS (Figure 3, D). DP of A1-A5 was close to 1 because they were located at 5’ end. DP of A6-A7 decreased and A7s had the minimal DP because they were close to 3’ end. Meanwhile they were not very close to 3’ end, so they were not affected by 3’ degradation (Figure 3, F). Surprisingly 11 probes of Os.4216.1.S1_a_at were located at the region of A7. Their locations were consistent with the fact that its intensity was higher in TCS than OCS. Several other genes also provided the similar results (See Additional file 1: Figure S4).

In conclusion, our Real Time PCR results indicated the correlation between position and intensity of probes in amplification processes: the probe close to the 5’ end would be underestimated because of degradation and random primer effect, while the probe very close to the 3’ end would be also underestimated because of 3’ degradation; the probe located but still had a distance to 3’ end might have the most exact expression measurement of probe set.

### The Coefficient of Variation of probe sets

**Figure 4:**
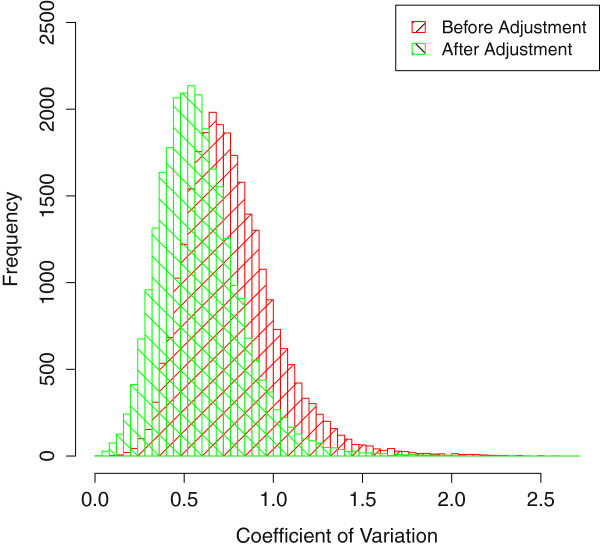
**Distribution of the Coefficient of Variation.** Distribution of the Coefficient of Variation (CV) for PM intensities of present probe sets before (Red) and after (Green) adjustment. The CV for probe sets decreased significantly after adjustment (p-value < 2.2e-16).

As probes of a same probe set measures the intensity of the same transcript, we expect the PM intensity of these probes to be more alike, i.e. have smaller Coefficient of Variation (CV). Using the microarray data generated with two cycle amplification in Data set 1, we calculated the CV for present probe sets before and after model adjustment, as shown in Figure 4. The histogram indicated that CV of these present probe sets decrease significantly after adjustment (p-value < 2.2e-16 with Wilcoxon Signed-Rank Test). Thus, the bias could be reduced efficiently and our model adjustment could preserve the inner structure of samples.

### Correlation of 15 rice stamen microarray samples

To further evaluate the biological meaning of our adjusting model, we created 21 rice microarray samples (Data Set 3,15 rice stamen samples, 3 leaf samples and 3 bud samples) using the Affymetrix two-cycle RNA labeling.

**Figure 5:**
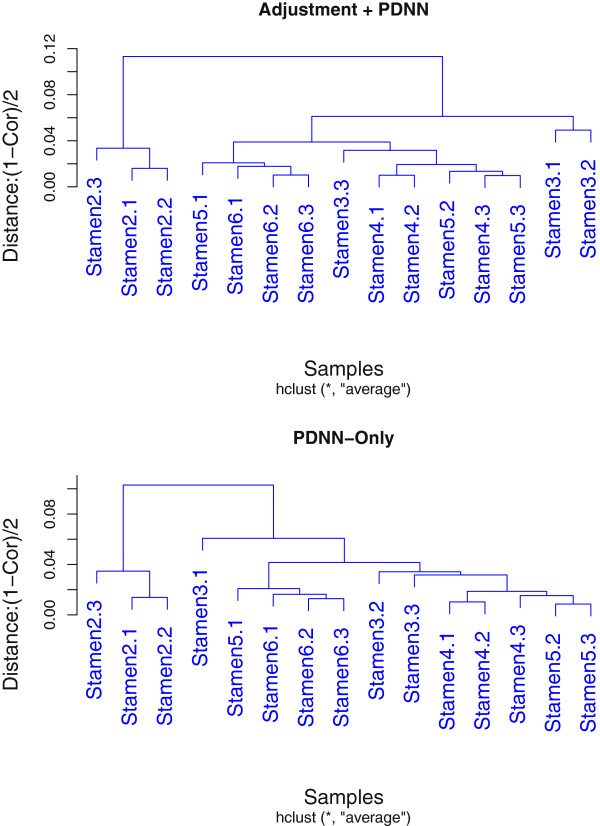
**Hierarchical clustering of stamen microarray samples.** Hierarchical clustering of 15 rice stamen microarray samples (PDNN) using 4234 probe sets only present in stamen samples. Left is clustering using PDNN preprocessed data with original PM, while right using PDNN preprocessed data with adjusted PM. Before adjustment, sample Stamen 3.1 is far away from the other two samples of stage 3. After adjustment, it is classified to be with sample Stamen 3.2.

**Figure 6:**
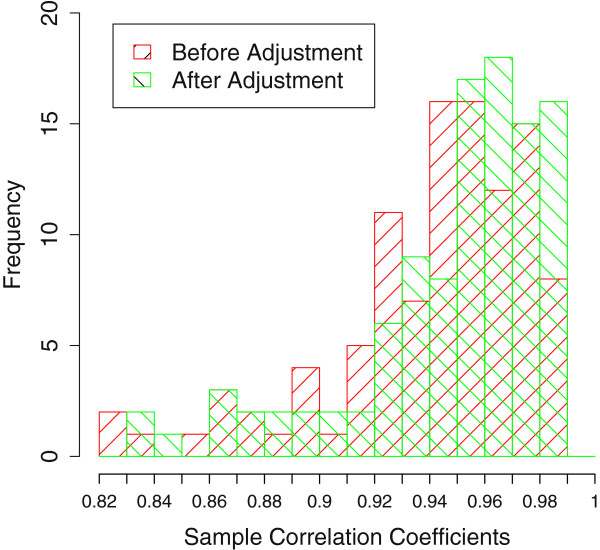
**Histogram of the correlation coefficient among 15 stamen micoarray samples.** Histogram of the correlation coefficient among 15 stamen micoarray samples before (Red) and after (Green) adjustment. There is an obvious increase after adjustment, which means the stamen samples are more alike, as we expected.

We first adjusted the PM intensity for 15 stamen microarray samples using our adjustment model, and then performed PDNN [15]. After that, we chose the probe sets only present in 5 stamen stages but not in leaf nor bud as a classifying set (Identified By MAS5.0, 4234 probe sets). The results of hierarchical clustering for the 15 stamen samples are shown in Figure 5. It shows that without adjustment, sample Stamen 3.2 is separate from other samples, while after adjustment, Stamen 3.1 is much closer to Stamen 3.2. Although Stamen 3.3 is far away, it is close to Stamen 4, thus samples of adjacent periods are classified closer, which is more biologically reasonable. In fact, the time of development from stamen stage 2 to stamen stage 6 is very short and is difficult to distinguish, thus makes samples abstracted from these stages to be very similar, i.e. the correlation efficient seems to be very high. Using the adjusted model, the correlation coefficients of samples (Using 28062 probe sets that are present in at least one of 5 stamen stages) increased significantly after adjustment (see Figure 6, p-value < 2.2e-16 with Wilcoxon Signed-Rank Test). These results demonstrated that we could get a more biologically reliable classification of samples after degradation adjustment.

## Discussion

Several works have considered the effect of mRNA degradation in microarray during the process of two-cycle linear amplification as well as the bias it caused [12], however, almost no or less work has been reported to establish adjusting methods to solve this problem.

In this study, focusing on the process of two-cycle linear amplification and the features of gene expression microarray data, we found two key factors that could bring biases: (i) Reverse transcription by random primers; (ii) RNA degradation in the first and second cycle amplification. We modeled these processes with uniform distributions and established a model to correct the bias. As we performed the adjustment for PM at probe level, one could further apply any kinds of microarray preprocessing methods, such RMA, dchip or PDNN to perform normalization. Note that we could not only apply the model to PM, but also to MM, as the Retaining Rate measures how probable each position of transcript remains after amplification, which has no relationship to binding mechanism. Other preprocessing methods considering MM could also be combined to do further reliable normalization.

To evaluate our model, we applied the adjustment method to microarray data of Data Set 1 and 3. From three aspects: (i) Coefficient of Variation for probes within a probe set decreases significantly, (ii)The clustering diagram shows more reasonable classification for rice stamen samples and (iii) the relationship among them become be much more closer, we could see that our model had obtained relatively more biologically reasonable results.

To demonstrate the necessity of our model for adjusting, we applied another adjusting method that assigning different weight to each position of transcript according to mean PM expression intensity. The main steps are designed as follows: (i) Plot the mean PM intensity for probes at 12 ∼588bp (probes in 98.68% of present probests) of transcripts. (ii) Apply lowess (locally weighted scatterplot smoothing) to fit the data. Then compute loess smoothed values for all points along the curve. Normalize all loess smoothed values to make their mean to be 1. Take the reciprocal of the normalized value at each position as the weight for probes at this position (See Figure S5). (iii) Adjust PM at each position by multiplying the PM intensity by the weight. (iv) Combine with known preprocessing methods (PDNN, or RMA). We call this process of adjusting Curve Adjustment (CA for short). To compare CA with our method, we applied both of them to Data Set 1 and 3. We could see from Figure S6 that, the CV didn’t decrease and the clustering of 15 samples were almost the same as that of none adjustment (Figure S7). Besides, the sample correlation coefficients didn’t raised much after CA (Figure S8). Thus, these results indicate that direct curve adjustment for microarray data is not suitable and our model adjustment is necessary. See Additional file 1 for more details.

## Conclusion

Although we could correct for the bias to some extent, there are still some challenges, such as the assumption of the distribution for random primer to be union, which may be different from actual condition. Besides, we applied a simple linear model to simulate RNA degradation and didn’t consider the different degradation rate caused by different nucleotides, such as GC-content, which may play an important role in two-cycle amplification. A new challenge will be the combination of both position of probe and sequence preference to make more accurate correction of bias.

With the development of Next Generation Sequencing, RNA-seq is widely utilized to measure gene expression at transcriptional level at unprecedented precision and throughput [20-24]. Our model may have further applications, as most RNA-seq Library Preparation Protocols also require RNA amplification. In this article, we just provide a basic idea of correcting for bias in microarray raw data to get more accurate result for further analysis, which may shed light on the adjusting methods for RNA-seq data.

Although the approaching of Next Generation Sequencing leads to more accurate results of gene expression, it is now still relatively too expensive. Microarray will still be a feasible way to measure gene expression in the near future. Our method could be applied to reuse the existing microarry data generated with two cycle amplification protocols, and the more biological promising results obtained by the adjusting model will surely benefit the following analysis, e.g. detecting differentially expressed genes or gene set analysis.

## Competing interests

The authors declare that they have no competing interests.

## Authors’ contributions

FW and DJ were responsible for the construction of the statistical model, FW and RC produced the manuscript, while RC and SB designed the microarray and Real Time PCR experiments and analyzed the Real Time PCR data. MD, SB and MQ provided essential suggestions for this work and helped to prepare the manuscript. All authors read and approved the final manuscript.

## Supplementary Material

Additional file 1 Supplemental Figures.
**Figure S1.** Position of probes on their transcripts (bp) (away from 3’ end) of Affymetrix GeneChip Rice Genome oligonucleotide arrays.
**Figure S2.** Correlation between position and intensity of probes for present probe sets (By MAS5.0) in Leaf and Leaf Primordium microarray data.
**Figure S3.** Schematic diagram of Real Time PCR experiments.
**Figure S4.** The Real Time PCR results for other transcripts show similar trends as in Figure 2.
**Figure S5.** Estimation of weight for curve adjustment.
**Figure S6.** Distribution of the Coefficient of Variation (CV) for PM intensities of present probe sets after 3 preprocessing methods.
**Figure S7.** Hierarchical clustering of 15 microarray samples after 3 preprocessing methods.
**Figure S8.** Histogram of correlation coefficients between 15 microarray samples after 3 preprocessing methods.
**Supplemental Formula.** Formula F1: The joint distribution for positions of the new 3 end and 5 end after the 3th shorten A3 and B3: F3(x, y).
**Supplemental Results and Discussion.** Comparison with Curve Adjustment to demonstrate the necessity of our model for adjusting bias. A simple adjusting method that assigns different weight to probes at different position of transcript according to expression intensity was applied, but the result indicates that direct curve adjustment for microarray data is not suitable and Model adjustment is necessary. Click here for file
