# Supplementary material for: Adjustment method for microarray data generated using two-cycle RNA labeling protocol
Source: BMC Genomics. 2013 Jan 16;14:31. doi: 10.1186/1471-2164-14-31 (PMC3658951; doi:10.1186/1471-2164-14-31)
Supplement: Additional file 1 — Supplemental Figures. Figure S1. Position of probes on their transcripts (bp) (away from 3’ end) of Affymetrix GeneChip Rice Genome oligonucleotide arrays. Figure S2. Correlation between position and intensity of probes for present probe sets (By MAS5.0) in Leaf and Leaf Primordium microarray data. Figure S3. Schematic diagram of Real Time PCR experiments. Figure S4. The Real Time PCR results for other transcripts show similar trends as in Figure 2. Figure S5. Estimation of weight for curve adjustment. Figure S6. Distribution of the Coefficient of Variation (CV) for PM intensities of present probe sets after 3 preprocessing methods. Figure S7. Hierarchical clustering of 15 microarray samples after 3 preprocessing methods. Figure S8. Histogram of correlation coefficients between 15 microarray samples after 3 preprocessing methods. Supplemental Formula. Formula F1: The joint distribution for positions of the new 3 end and 5 end after the 3th shorten A3 and B3: F3(x, y). Supplemental Results and Discussion. Comparison with Curve Adjustment to demonstrate the necessity of our model for adjusting bias. A simple adjusting method that assigns different weight to probes at different position of transcript according to expression intensity was applied, but the result indicates that direct curve adjustment for microarray data is not suitable and Model adjustment is necessary. [file 1471-2164-14-31-S1.doc]

Additional files for:

Adjustment method for microarray data generated using two-cycle RNA labeling protocol

Fugui Wang^1*^, Rui Chen^3*^, Dong Ji^1,2^, Shunong Bai^3^, Minpin Qian^1,2^, Minghua Deng^1,2^

^1^Center for Quantitative Biology, Peking University, Beijing, 100871, China.

^2^School of Mathematical Sciences, Peking University, Beijing, 100871, China.

^3^School of Life Science, Peking University, Beijing, 100871, China.

I. Supplemental Figures


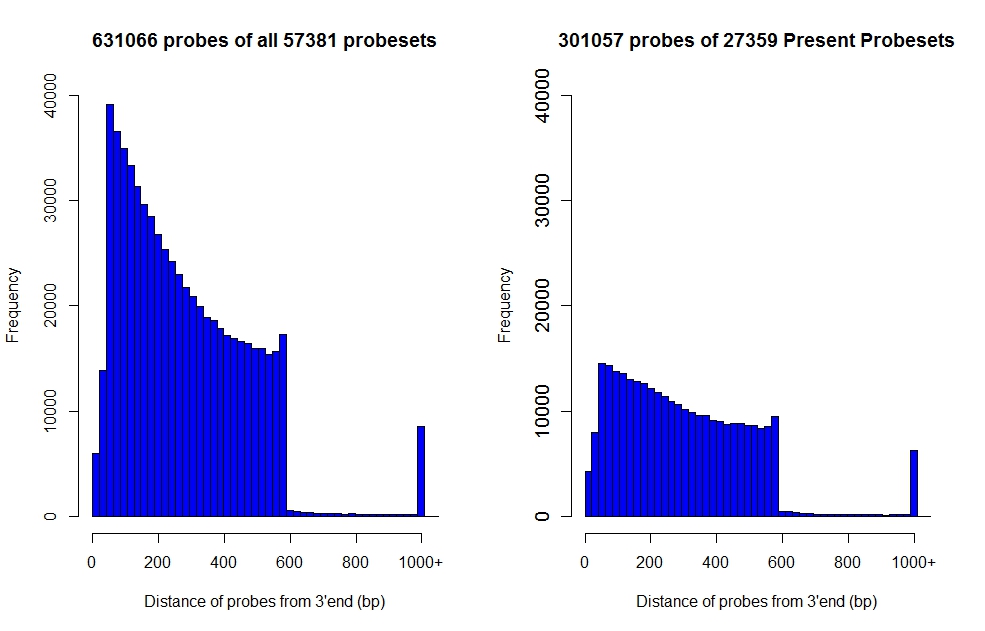


A

B

**Figure S1. Position of probes on their transcripts (bp) (away from 3’end) of Affymetrix GeneChip Rice Genome oligonucleotide arrays.** (A) With 631066 probes of all 57381 probesets, it obviously demonstrates that most probes are designed to have distances less than 600 bp from 3’end of transcripts. That’s why we treat 3’end as the start of x-axis in our study. The probes that have distance larger than 1000 bp account for about 1.33% of total 631066 probes (B) 301057 probes of present probesets (Detected by mas5calls in MAS5.0). The trend is almost the same as that for all 631066 probes.

**
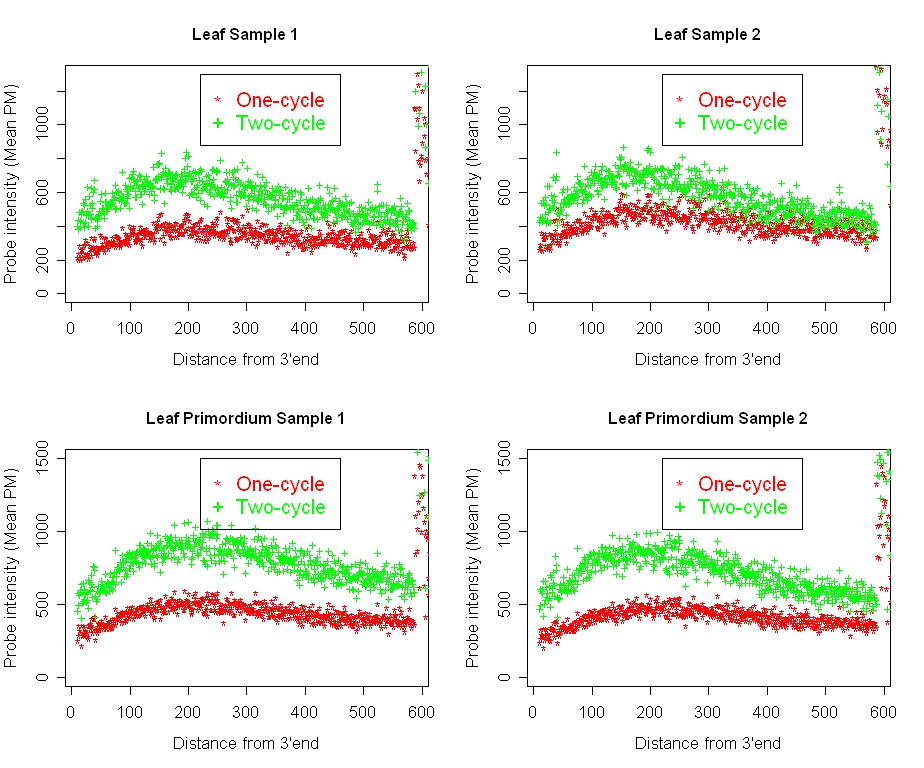
**

**Figure S2. Correlation between position and intensity of probes for present probe sets (By MAS5.0) in Leaf and Leaf Primordium microarray data.** The data was generated with one-cycle and two-cycle labeling protocols (Data Set 2). These four replicates show similar degradation trends and the bias in two-cycle is more severe.


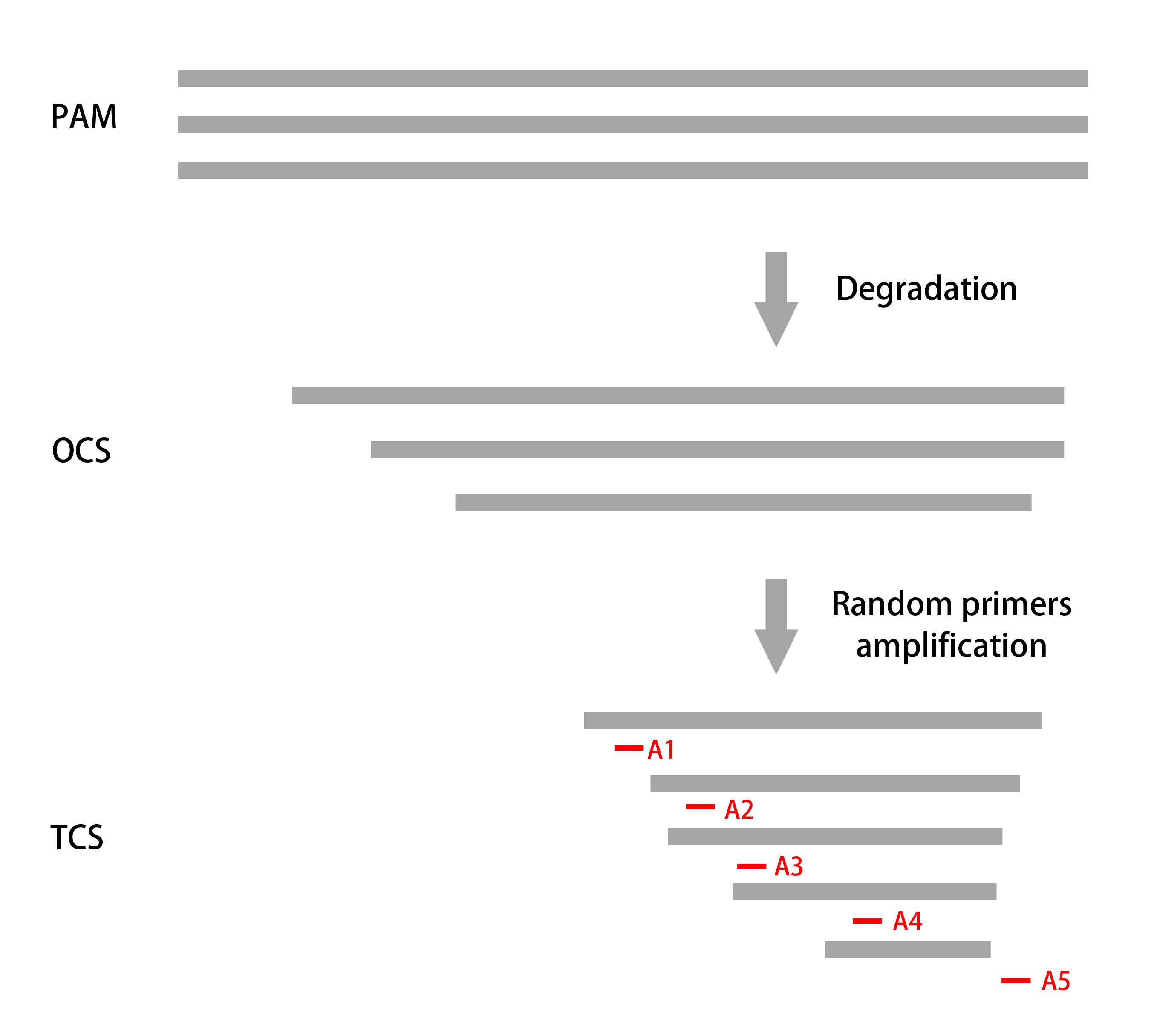


**Figure S3.** **Schematic diagram of Real Time PCR experiments.** In PAM, a gene’s transcripts are subequal. After one-cycle amplification, some transcripts become shorter on both ends. Because of amplification using random primers in two-cycle amplification, most of transcripts are shorter further. Because amplicons were designed at different positions, so they could detect transcripts with different length. In this figure, A4 amplicon have the MAX(R_ij_) and could represent the most transcripts in the sample, so we could choose the maximum intensity to approximatethe true intensity of transcripts. PAM, Pre-amplified mRNA samples; OCS, One-Cycle cRNA samples; TCS,Two-Cycle cRNA samples; A1-A5, amplicon 1-5.


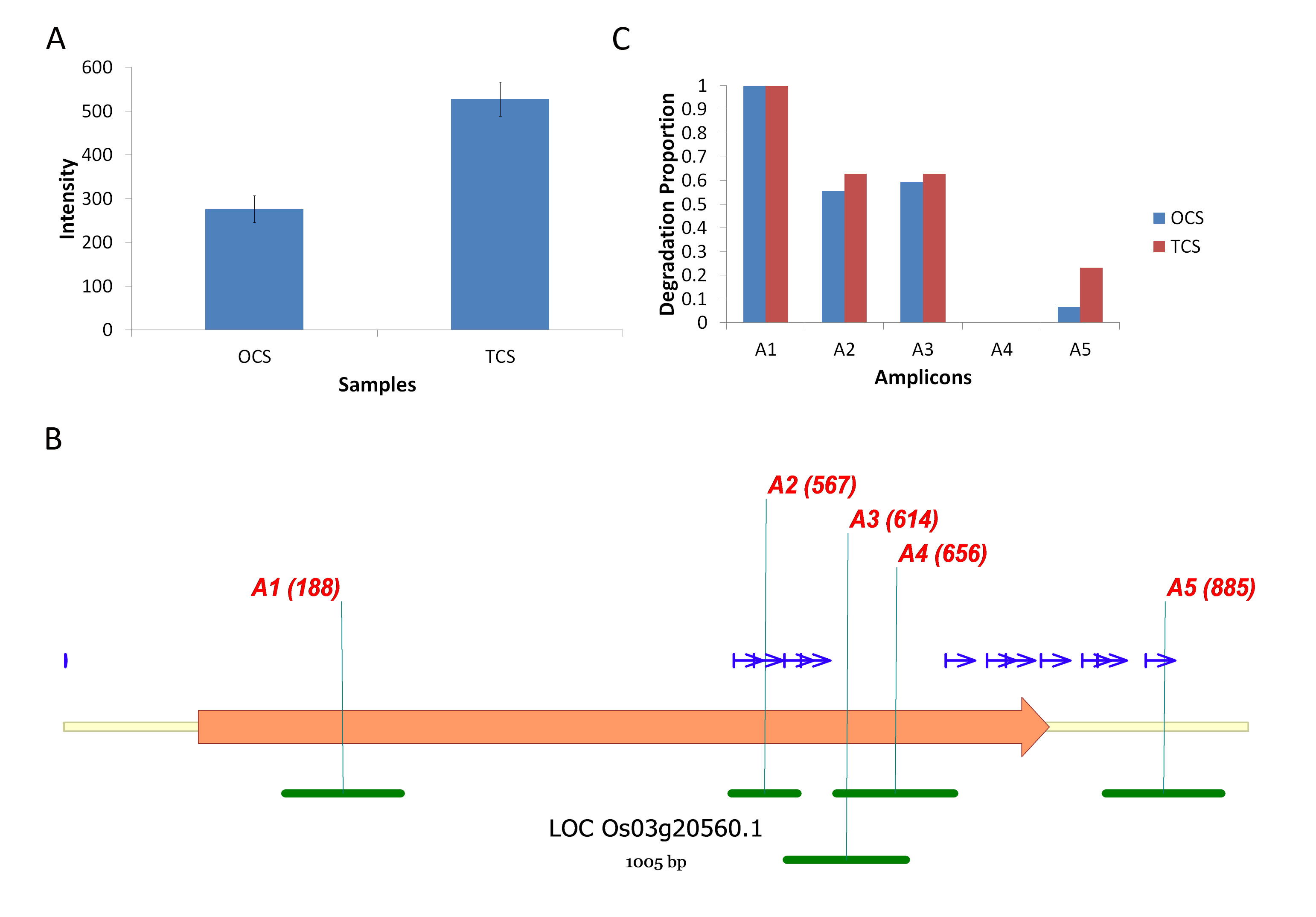

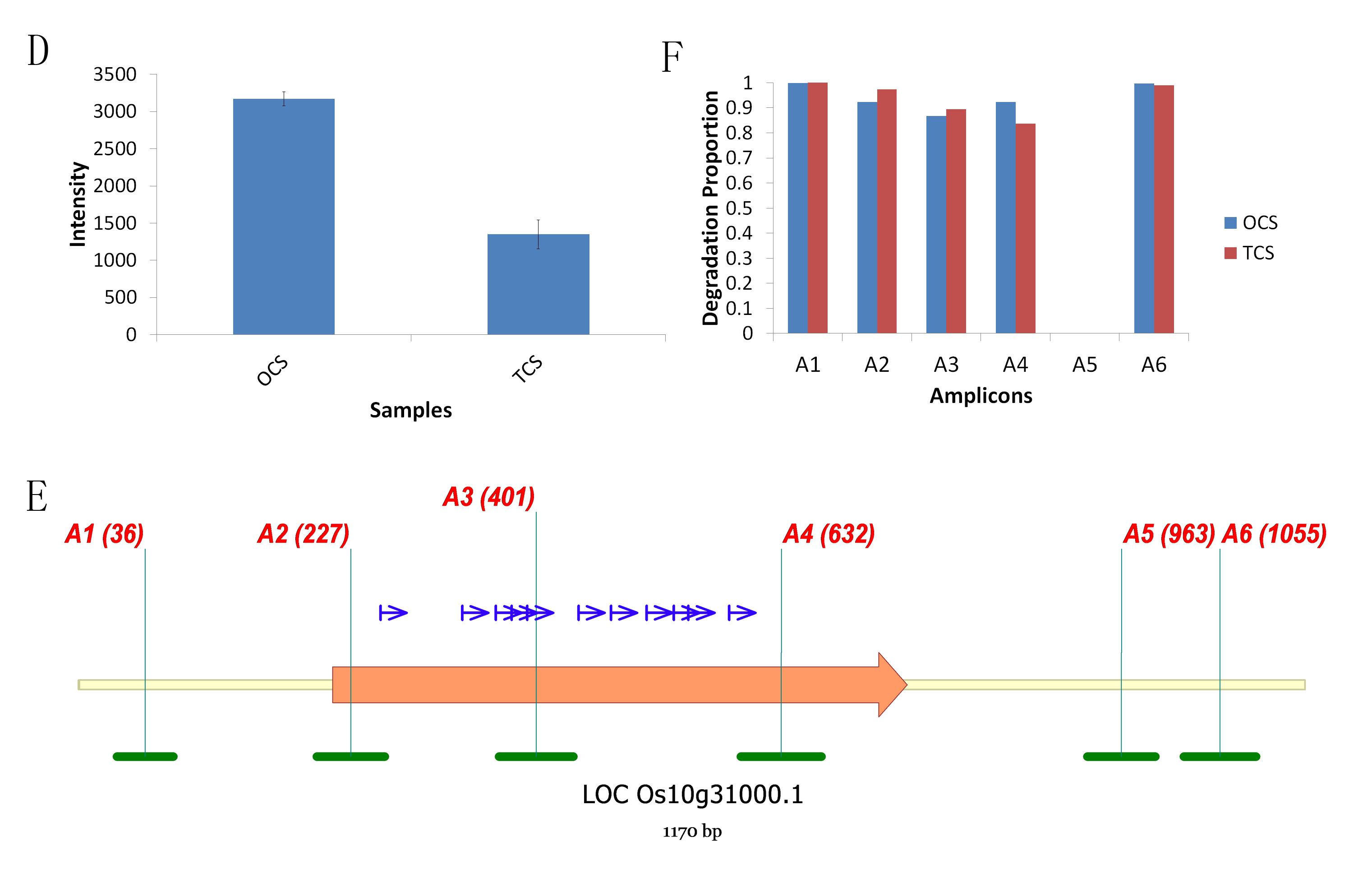


**Figure S4. The Real Time PCR results for other transcripts show similar trends as in Figure 2.** (A-C) Data for LOC_Os03g20560.1. (A) The intensity of LOC_Os03g20560.1 increased dramatically in Two-Cycle cRNA sample (TCS) comparing with One-Cycle cRNA sample (ONS) in our microarray experiment. (B) Schematic diagram of LOC_Os03g20560.1. Yellow line, cDNA of the gene; big orange arrow, coding sequence (CDS) of the gene; Green line section (A1-A6), designed amplicons in Real Time PCR experiments; Numbers in the brackets were the starting point of amplicons from 5’ end; Blue narrow arrow, designed probes on the microarray. Their starting points (unlabeled in the figure) were 565, 582, 608, 622, 745, 780, 796, 826, 861, 874, and 915. (C) Degradation Proportion (DP, see details in methods section) of amplicons (A1-A7) showed in (B). DP decreased along with distance from the 5’ end and increased when it was too close to 3’ end because of degradation and random effect. The probes on microarray are designed to be within A2~A5, where the DPs are relatively small, which can explain why the intensity of the probeset increased after TCS. (D-F) Data for LOC_Os10g31000.1. (D) The intensity of LOC_Os10g31000.1. (E) Schematic diagram of LOC_Os10g31000.1. The probes’ starting points were 262, 340, 372, 387, 402, 451, 482, 516, 542, 556, and 595. (F) DP of amplicons. DP decreased along with distance from the 5’ end and increased. The probes on microarray are designed to be within A2~A4, where the DPs are very high, which can explain why the intensity of the probeset decreased after TCS.

II. Supplemental Formula

**Formula F1:** For a given transcript t, whose length is L (bp), the degrading limits for 3' end and 5' end is a and b, while the new 3' end and 5' end after the *3rd* shorten are *A_3_* and *B_3_*, respectively. The joint distribution of positions for is *F_3_(x, y)*, here *F_3_(x, y)* could be written out as:

**III.** Supplemental Results and Discussion

**Comparison with Curve Adjustment**

To demonstrate the necessity of our model for adjusting bias, we compared a simple adjusting method that assigns different weight to probes at different position of transcript according to expression intensity. The adjusting process is shown as follows:

**(i)** Plot the mean expression intensity for position at 12~588bp (probes in 98.68% of present probests) of transcript. (See Fig.S5)

**(ii)** First apply *lowess* (locally weighted scatterplot smoothing) to fit the data. Then compute loess smoothed values for all points along the curve. Normalize all loess smoothed values to make their mean to be 1. Take the reciprocal of the normalized value at each position as the weight for probes at this position. (See Fig.S5)

**(iii)** Adjust PM at each position by multiplying the PM intensity by the weight.

**(iv)** Combine with known preprocessing methods (PDNN, or RMA).

We call this process of adjustment ***Curve Adjustmen***t.

To compare ***Curve Adjustmen***t with our method (***Model adjustment***), we applied both of them to ***Data set 1* and *Data set 3*** (more details see Materials and Method section). We could see from Figure.S6 that, there is a significant decline for Coefficient of Variation (CV) for PM intensities of present probe sets after ***Model adjustment****,* while it almost didn’t change after ***Curve Adjustmen*t**. Figure.S7 shows that the clustering of 15 samples after ***Curve Adjustmen***t are almost the same as that of none adjustment. Besides, the sample correlation efficiencies didn't raised much after ***Curve Adjustmen*t** (Figure.S8), while there is a remarkable increase after ***Model adjustment.*** Thus, these results indicate that direct curve adjustment for microarray data is not suitable and ***Model adjustment*** is necessary.

**
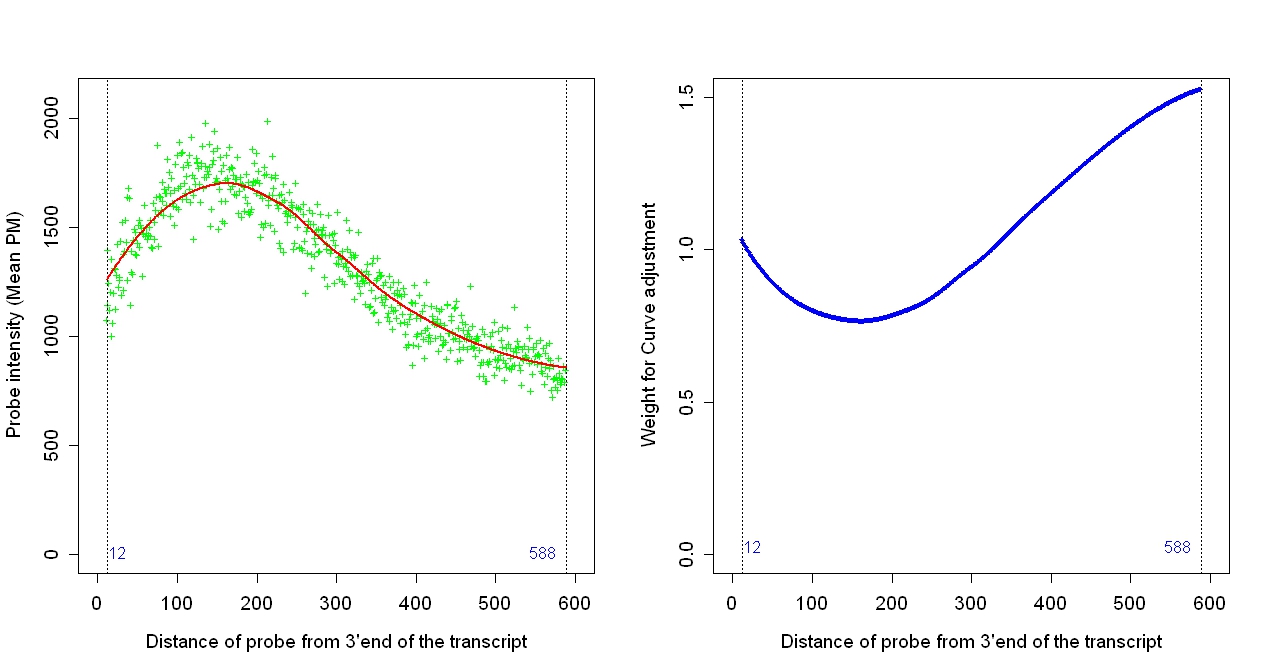
**

A

B

**Figure S5. Estimation of weight for curve adjustment.** (A)The correlation between position and intensity of probes for present probe sets (By MAS5.0) in two-cycle amplification microarray data of Data Set 1. x-axis is the distance of probe from 3'end of its corresponding transcript, while y-axis is the mean PM intensity of probes at each position. Red curve is the lowess fitted curve. (B) The weight for probes at position 12bp~588bp of transcript in curve adjustment.

**
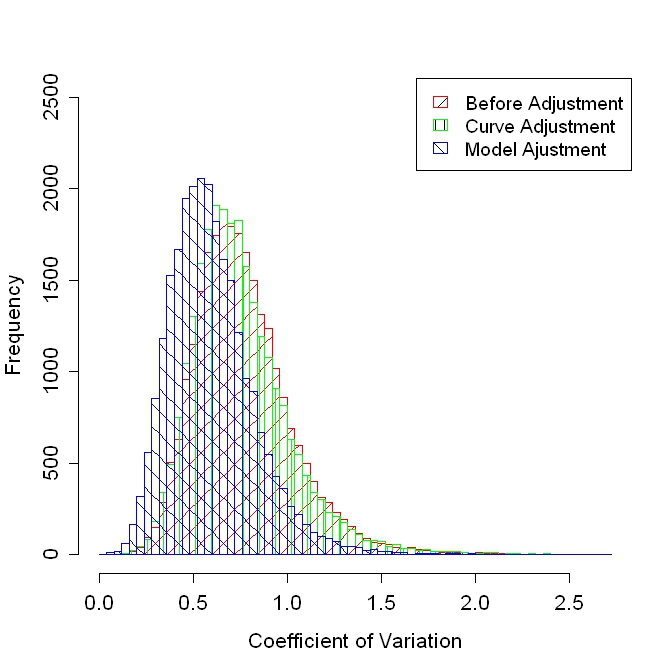
**

**Figure S6. Distribution of the Coefficient of Variation (CV) for PM intensities of present probe sets.** Before (Red), Curve Adjustment (Green) and Model adjustment (blue). There is a significant decline after Model adjustment.


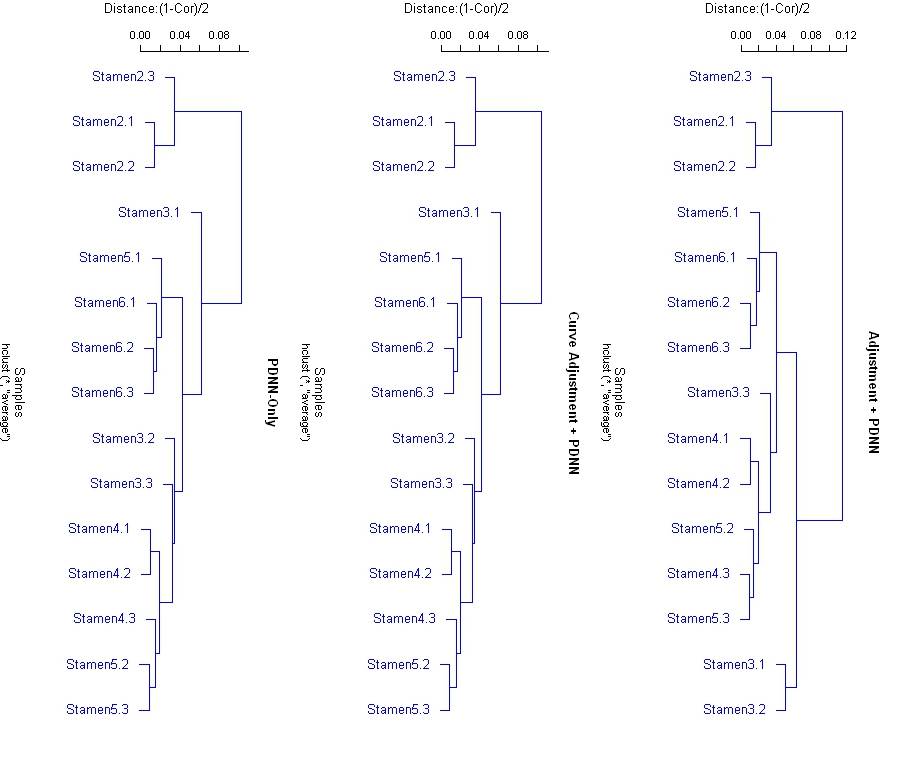


**Figure S7. Hierarchical clustering of 15 microarray samples.** Before adjustment (left), after Curve Adjustment (Middle) and after Model Adjustment (Right) (PDNN, 4093 probesets only present in stamen and have probes all within 12~588bp). The clustering results showed that neither before nor after Curve Adjustment could separate Stamen sample 3.1.

**
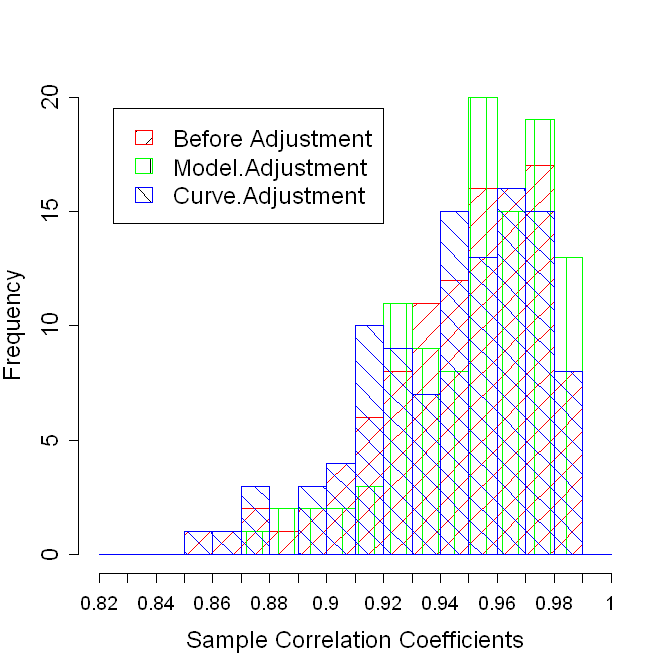
**

**Figure S8. Histogram of correlation coefficients between 15 microarray samples (Data Set 3) before and after adjustment.** We cloud see that our Model adjustment increase significantly than both non- and curve- adjustment.
